# Development, acceptability and feasibility of a communication skills training package for therapeutic radiographers to reduce fear of recurrence development in breast cancer patients (FORECAST2)

**DOI:** 10.1186/s40814-018-0338-9

**Published:** 2018-09-18

**Authors:** Mara van Beusekom, Josie Cameron, Carolyn Bedi, Elspeth Banks, Tom Kelsey, Gerry Humphris

**Affiliations:** 1 School of Medicine, University of St Andrews, St Andrews, UK; 2 Edinburgh Cancer Centre, Western General Hospital, Edinburgh, UK; 3 National Cancer Research Institute, London, UK; 4 School of Computer Science, University of St Andrews, St Andrews, UK

**Keywords:** Communication, Emotional regulation, Fear of cancer recurrence, Breast cancer, Co-design, Acceptability

## Abstract

**Background:**

Many patients who have been treated for breast cancer experience high levels of fear that the cancer will return. The FORECAST pilot study showed that for a third of the patients, fears of cancer recurrence (FCR) increase during radiotherapy treatment and that conversations with their therapeutic radiographer at the weekly review meetings might help patients manage these concerns. This study aims to develop a communication skills training package (KEW, for ‘Know’, ‘Encourage’ and ‘Warm-up’) for therapeutic radiographers based on the findings of the FORECAST pilot study and on active input from patients and radiographers. This package will be piloted in a single centre to evaluate its acceptability and to prepare for a multi-centre clinical trial.

**Methods:**

The study consists of three phases. In the first phase, patient representatives and therapeutic radiographers participate in Experience-Based Co-Design to identify ways to improve communication during the radiotherapy review. In the second phase, various stakeholders, including members of the Society of Radiographers and of national patient representation groups, are consulted to develop a storyboard for the production of the communication training package. In the third phase, the acceptability and feasibility of the training is evaluated through observations, recruitment rates and follow-up discussions; a fidelity measure is designed; and potential benefits are observed, with patients’ fear of cancer recurrence (FCR7) as the primary outcome. Secondary outcomes include a short daily measure of recurrence (FCR3), patients’ positive and negative affect (PANAS), perceived empathy from the radiographer (CARE), satisfaction with the review meetings (RISS) and health-related quality of life (EQ-5D-3L).

**Discussion:**

To date, there has been limited research on how communication between therapeutic radiographers and patients during review appointments can help to manage patients’ recurrence fears during radiotherapy treatment. A collaborative and participatory approach to the development of a communication skills training will ensure that it is optimally targeted to the needs and preferences of both patients and radiographers. Targeting recurrence fears through communication at this stage, when patients are still in regular contact with healthcare providers, has the potential to reduce the need for complex interventions post-treatment.

**Trial registration:**

NRES reference: 18/LO/0669. Clinicaltrial.gov ID: NCT03468881

## Background

Breast cancer is the most common cancer in the UK, with 54,900 new cases every year [1]. Steadily improving survival rates also mean that the number of people who are breast cancer survivors is growing [2]. After treatment has finished, patients’ physical and psychological symptoms do not immediately end and survivors need to be supported to cope with the consequences of their treatment and with the concerns that they experience [3]. Often, breast cancer survivors are most concerned about the possibility of their cancer returning [4–6]: up to 86% of all breast cancer survivors experience some level of this fear, moderate levels are reported by 24 to 56%, and up to 14% of survivors experience high levels of recurrence fears [7].

Fear of cancer recurrence (FCR) has been defined as the ‘fear, worry, or concern relating to the possibility that the cancer will come back or progress’ [8]. High levels of FCR are associated with higher health costs [9] and have been linked to health behaviour and psychological responses such as distress, depression and anxiety [7]. Due to its high prevalence and serious consequences, it has been advised to carry out routine assessment for FCR following treatment [9]. To help patients who do experience clinical levels of recurrence fears, various psychological interventions have been developed, including Conquer Fear [10], SWORD (‘Survivors’ Worries of Recurrent Disease’) [11] and the AFTER intervention [12].

However, ideally, patients would be supported in such a way to minimise the development of these fears before they rise to clinical levels, which was the focus of the FORECAST pilot study [13]. The Lee-Jones model of FCR describes that in addition to patients’ internal cues, external cues such as communication with healthcare providers can trigger an emotional and cognitive response that result in patients’ personal perception of recurrence [14]. A recent study also found empirical evidence for the relation between such cues and FCR [15]. In addition, there is work that suggests that patients’ perceived quality of interactions during the diagnostic consultation can indeed affect their FCR levels [16].

FORECAST analysed conversations between 89 patients and their radiographers during the weekly review appointments and identified these sessions as a crucial target point—it was seen that patients whose FCR trajectories decreased during radiotherapy were more likely to mention cancer directly at the review appointments, had longer consultations, and expressed twice as many emotional concerns [17]. Furthermore, a significant relationship was seen between patients FCR levels and closing down of patients’ conversations in a clinical encounter during a review appointment in the radiotherapy unit. Since radiotherapy services for breast cancer patients often include a review service [18], this could be an optimal moment to address patients’ recurrence fears before they rise to excessive levels. The outlined protocol describes the follow-up to this study, FORECAST2 [19]. FORECAST2 builds upon the findings of the pilot study to develop a communication skills training package for therapeutic radiographers, to help them respond to emotional concerns that are expressed by patients at the review appointments. Theoretically, the package will be modelled on an intervention for general practitioners that successfully helped patients feel less distressed and more satisfied with the consultation [20] to target patient *Knowledge*, *Encouragement* to patients to express concerns and *Warm-Up* to avoid sudden cutoffs at the end of the conversations (KEW). In addition, patients and therapeutic radiographers will be actively involved in the development of the training package through *Experienced-Based Co-Design* (EBCD), to encourage the exchange of experiences between patients and healthcare providers and identify shared points for improvements and solutions [21].

The aims of the study outlined in this protocol are (1) to develop a communication skills training package for therapeutic radiographers and (2) to pilot this package in a single service. The evaluation will include the observation of potential benefits in practice, in particular the effect of the package on patients’ development of FCR trajectories. In addition, the single-service evaluation will help to prepare for a formal randomised trial by evaluating the acceptability and feasibility of presentation to staff and patients, completing electronic diary assessment of recurrence fears, and the design of a fidelity measure.

## Methods/design

**Fig. 1:**
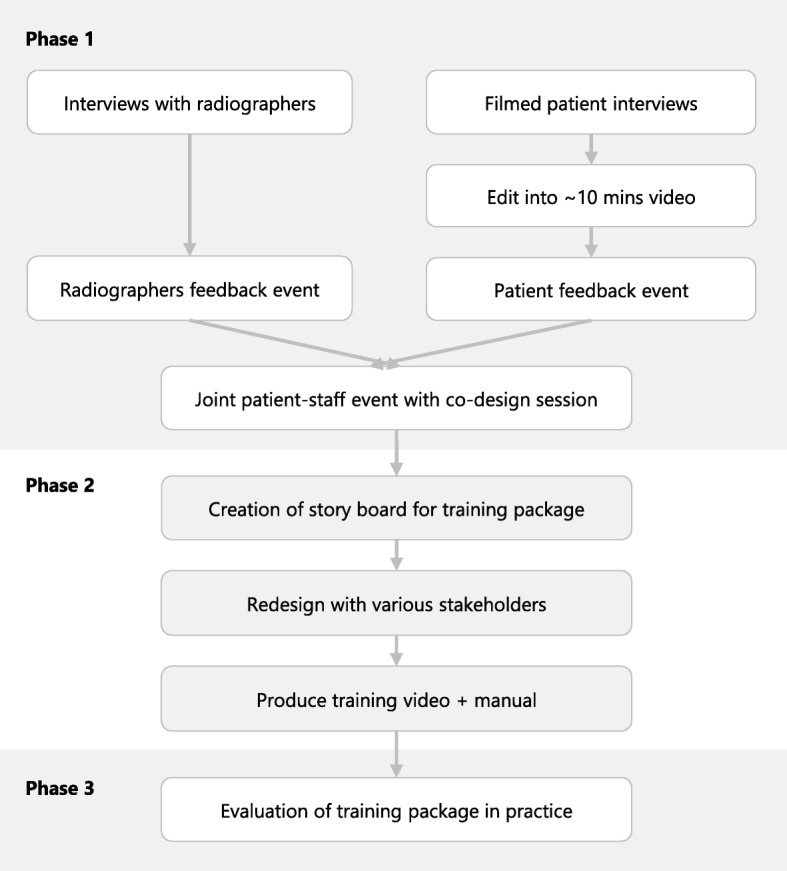
Flowchart of outlined study, consisting of three consecutive phases

This study consists of three phases (Fig. 1): a co-design, production and evaluation phase.

### Phase 1—Co-design

The aim of the first phase is to identify shared issues in communication during radiotherapy treatment with breast cancer patients and therapeutic radiographers and to work on solutions to help improve this communication. This exploratory phase of the study will be guided by principles of EBCD [22] and will make use of a variety of participatory methods, including card sorting to identify priorities, brainstorming to generate ideas and the emotional mapping exercise [23], in which patients can identify and rank critical moments in their radiotherapy treatment. Four patient representatives and three therapeutic radiographers will be recruited at a specialist breast cancer centre in Scotland through the investigators’ networks, based on willingness to participate. Eligible patient representatives are female, are over 18 years of age and have received radiotherapy to treat their breast cancer.

Phase 1 will start with introductory individual interviews with the patients and radiographers to identify their experiences with the radiotherapy service and to discuss improving communication (Table 1). The patient interviews will be filmed, and a short video will be edited to capture patient experience. This film will be validated at a patient feedback event and later shown at a joint patient-staff event as the starting point to share experiences and agree on areas for improvement. Prior to the joint event, radiographers will also have a feedback event to validate findings from the interviews and to distinguish between issues to be addressed internally and those to be discussed in the co-design process. After the video is discussed at the joint patient-staff event, a co-design session follows to work on solutions to the issues that are raised. All sessions that are not filmed will be audiotaped and can be transcribed to identify themes in participants’ responses using a thematic framework approach; emphasis will be on summarising and validating participants’ input during the sessions.

**Table 1:** Topics discussed during sessions of phase 1

Interview radiographers	
Background about role	
Staff experiences	
Perceptions of patient experience	
Improving communication	
Feedback event radiographers	
Validation of findings from interview	
Distinguish between topics to be addressed ‘internally’ and for co-design	
Interview patients	
Journey so far	
Satisfaction with radiotherapy experience	
Crucial points in radiotherapy process	
Improving communication	
Feedback event patients	
Validation of findings presented in video	
Mapping and rating of key stages and moments; agree on topics to be discussed with staff	
Joint staff-patient event	
Exchange experiences (using video)	
Determine shared priorities	
Brainstorm to identify solutions	

### Phase 2—Production

The second phase of the outlined study works towards the production of a video and training manual for the communication training package. Based on the findings of phase 1, evidence on successful strategies to improve patients’ satisfaction with consultations [20] and on correlations between patient-provider interactions during review appointments and patients’ levels of FCR [17], the modules that will be covered in the training workshop will be determined and a storyboard will be created for the production of the training package. Examples of the types of communication interventions that are likely to be covered include open-ended general questions on how the patient is managing and how they are feeling, to listen to enquiries and provided additional information (*Knowledge*), to acknowledge emotional cues and prevent closing the patient down (*Emotion*) and to give a positive finishing statement on closing the review session (*Warmth*). The storyboard will be evaluated and adjusted in an iterative process with consultative input from a variety of stakeholders.

Participants in this phase will include up to ten members of patient representation groups throughout the country and a radiotherapy stakeholder group with eight members of the Society of Radiographers. Participants will be recruited through the networks of the authors based on willingness to participate. Inclusion criteria for patient participants are as described for phase 1 of the study. Based on the logistical feasibility, consultative input will be invited either through face-to-face interviews or through email, using the Feedback Capture Grid (Interaction Design Foundation). Face-to-face sessions will be audiotaped, and themes in participants’ responses will be explored as described for phase 1.

### Phase 3—Evaluation

The aim of the third phase of the study, an observational cohort study, is to evaluate the communication training package in a specialist breast cancer centre in Scotland. All women with breast cancer who undergo radiotherapy treatment at this centre are seen at the weekly review appointments (usually two to three times and up to four to five for patients with ductal carcinoma in situ), as follow-up appointments and GP letters are generated at these clinics. Therapeutic radiographers who are willing to participate will follow an in-person session with a trained facilitator using the communication skills training package. Written materials will be available with examples, and workshop experiential sessions will be provided, as well as supervision by a clinical psychologist and communication trainer.

**Fig. 2:**
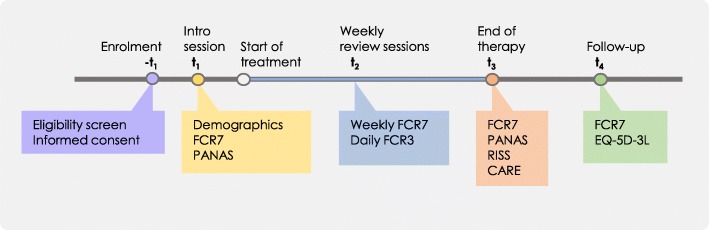
Timeline of study enrolment and assessments of the evaluation phase (phase 3): after SPIRIT [34]

The radiographers will screen for patient eligibility (i.e. adult, English-speaking, female invasive breast cancer patients about to receive radiotherapy as primary or adjuvant treatment) and invite patients to participate in the study (t_−1_) (Fig. 2). Patients who are interested to participate are referred to the investigator who will provide them with more information. Written informed consent is obtained from those who are willing to participate. Around the radiotherapy planning session (t_1_), patients are asked to provide their demographic details and to fill out the seven-item Fear of Cancer Recurrence (FCR7) measure and Positive And Negative Affect Scale (PANAS) (Table 2). During radiotherapy treatment, the regular weekly review consultations between the patient and therapeutic radiographer will be audiotaped (t_2_). During these 3 to 4 weeks, patients fill out FCR7 on a weekly basis and the three-item Fear of Cancer Recurrence (FCR3) on a daily basis, either in the form of a printed diary or in a smartphone application according to patient preference. At the end of radiotherapy (t_3_), patients fill out the FCR7 and PANAS again and evaluate their experience with the consultations using the Consultation and Relational Empathy (CARE) measure and the Radiotherapy Interview Satisfaction Scale (RISS), a version of the Medical Interview Satisfaction Scale modified to better fit the radiotherapy consultations. Six to eight weeks after the end of their treatment (t_4_), patients will receive a telephone call from the investigator to measure their recurrence fears (FCR7) one last time and their health status, using the EQ-5D-3L.

**Table 2:** Instruments used in evaluation of patient outcomes and psychometric properties

Instrument	Measures	Properties
FCR7	7-item self-report measure of patients’ Fear of Cancer Recurrence	Internal consistency: Cronbach’s *α* of 0.92. Construct validity: *r* = 0.68 versus Anxiety Sub-scale of Hospital and Depression Scale [35]
FCR3	3-item self-report measure of patients’ Fear of Cancer Recurrence	Based on the 4-item scale FCR4, with internal consistency: Cronbach’s *α* of 0.93. Construct validity: *r* = 0.65 versus Anxiety sub-scale of Hospital and Depression Scale [35]
PANAS	20-item self-report measure of Positive And Negative Affect	Internal consistency: Cronbach’s *α* of 0.89 for Positive Affectivity; 0.85 for Negative Affectivity. Construct validity: *r* = 0.52 versus HADS depression sub-scale; *r* = 0.67 versus Depression Anxiety and Stress Scales (DASS) stress sub-scale and 0.65 versus HADS anxiety sub-scale [36]
RISS	8-item self-report measure of patients’ Radiotherapy Interview Satisfaction	Adapted from the Medical Interview Satisfaction Scale (MISS-21) with internal consistency: 0.67–0.92 for sub-scales. Construct validity: *r* = 0.21–0.63 versus aspects of satisfaction with consultations [37]
CARE	10-item self-report measure of patients’ experience of Consultation and Relational Empathy	Internal consistency: Cronbach’s *α* of 0.92. Convergent validity: *r* = 0.85 versus Reynolds Empathy Measure (RES) and sufficient face validity [38]
EQ-5D-3L	5-item self-report measure of patients’ health status relating to mobility, self-care, usual activities, pain and discomfort, and anxiety and depression	Well-established reliability and validity; used in many countries and for many different conditions [39, 40]

In addition to the observation of potential benefits relating to patient outcomes, the acceptability and feasibility of the communication training package is evaluated through analysis of the audiotaped sessions and follow-up discussions with patients and radiographers. A 10% sample of patients will be invited to give free response comments from semi-structured interviews to their experience of the review consultations. These patients will be prompted to state the acceptability (rating on a 1 to 5 ordinal scale from ‘not acceptable’ to ‘completely acceptable’) of the major elements of the intervention, e.g. staff instructed to give patient space to expand on a concern. Prompts will cover the various dimensions of ‘acceptability’ including content, complexity and comfort [24]. All staff will be invited to give a similar acceptability rating for each element and an overall score of the intervention. Free responses will be analysed using a thematic framework approach. To help prepare for a multi-centre randomised controlled trial, participant recruitment and retention rates will be calculated and a fidelity measure will be designed to evaluate the extent to which the training delivery and implementation of the trained communication skills follows the designed protocols.

### Data management

Data from paper questionnaires will be anonymised by replacing the participant’s name with a unique identifying code and saved in a password-protected database. Data will be coded and entered into SPSS 24. Storage and analysis will take place in a ‘safe haven’ at the University, a room with secure entry procedures dedicated to analysing audio-visual materials. The key with names and ID numbers will be stored in a secure location at the study site. After audiotaped conversations are transcribed and coded, all uniquely identifying information will be blanked out. Data that is collected through the mobile phone application will be visible for participants (via password-protected access to the mobile application to guard against view by third parties with access to the device) and sent to a storage server in the ‘safe haven’ using Transport Layer Security (version 3) when the participant’s phone is connected to WIFI or mobile phone signal. An AES 256-bit double-layer encryption system [25] will be used to store the data.

### Analyses

Audiotaped sessions from phases 1 and 2 will be transcribed and content analysed [26], using a hybrid strategy of inductive and deductive thematic analysis [27]. Consultations that are audiotaped in phase 3 will be analysed using the Verona coding definitions of emotional sequences (VR-CoDES) [28], as described in the FORECAST pilot study [17]. Latent variable growth curve analysis (MPLUS) will be used to determine patients’ FCR trajectories and link these to FCR7 outcomes. Patient outcomes from the outlined study, in which therapeutic radiographers have been exposed to the communication skills training package, will be compared to patient outcomes from the pilot study that took place prior to the introduction of the training package. The cohort of the FORECAST pilot study was drawn from the same institution; any differences will be controlled for statistically in a conventional method of covariate inclusion. Matching the sample size of the pilot study cohort, the aim is to recruit 74 patients so that an effect of 0.375 (medium to low) can be detected at 80% power with a two-sided alpha of 0.05, to evaluate the effect of the training package on FCR levels.

## Discussion

The outlined protocol describes the development of a communication skills training package for therapeutic radiographers and an evaluation of its acceptability and feasibility in a single centre. The findings of the FORECAST pilot study indicate that patients’ review sessions with their radiographers during radiotherapy treatment may indeed be a good moment to address patients’ concerns to minimise the development of excessive recurrence fears [17]. The KEW training package aims to support therapeutic radiographers with recommendations and structured responses to concerns expressed by patients.

Based on previous findings and a successful intervention in the primary care setting [20], three facets of the training programme will be to target patient knowledge, to encourage patients to express their concerns and to avoid cutoffs at the end of the conversation. In addition, we will ask for participatory input from both patients and therapeutic radiographers by making use of principles from the EBCD method and ask other patient representatives and specialists in radiotherapy for consultative feedback when developing the training package. While there are some studies that have started to consider communication skills training with members of the radiotherapy team [29–31], none of these have involved patients in the design process of the programme.

EBCD has proven to be helpful to encourage patient engagement and obtain a more comprehensive consultation [32]. At the same time, there are some challenges that users of the method may encounter: the process can be experienced as complicated, it can be difficult to get staff engaged with the project and it is quite time-consuming [33]. It will therefore be essential to involve staff from the onset of the study and communicate carefully about expectations to all participants. Group sizes have been decreased to shorten the duration of the sessions. As there will only be one co-design event, it will also be the key to encourage active engagement in this session and to avoid it turning into ‘token participation’.

The study is expected to lead to new insights on how to support radiographers with emotional conversations and help patients manage their concerns about cancer recurrence, and prepare us for a multi-centre roll-out and randomised controlled trial to evaluate the resulting communication package.

## Abbreviations

AES: Advanced Encryption Standard
CARE: Consultation And Relational Empathy measure
EBCD: Experienced-Based Co-Design
EQ-5D-3L: Three-level version of EQ-5D instrument
FCR: Fear of cancer recurrence
FCR3: Three-item Fear of Cancer Recurrence Scale
FCR7: Seven-item Fear of Cancer Recurrence Scale
PANAS: Positive And Negative Affect Scale
RISS: Radiotherapy Interview Satisfaction Scale
VR-CoDES: Verona coding definitions of emotional sequences
